# Design of a prospective, multicenter, global, cohort study of electromagnetic navigation bronchoscopy

**DOI:** 10.1186/s12890-016-0228-y

**Published:** 2016-04-26

**Authors:** Erik E. Folch, Mark R. Bowling, Thomas R. Gildea, Kristin L. Hood, Septimiu D. Murgu, Eric M. Toloza, Momen M. Wahidi, Terence Williams, Sandeep J. Khandhar

**Affiliations:** Beth Israel Deaconess Medical Center, Harvard Medical School, Boston, MA USA; Department of Internal Medicine, Division of Pulmonary Critical Care and Sleep Medicine, Brody School of Medicine, East Carolina University, Greenville, NC USA; Department of Pulmonary, Allergy, and Critical Care Medicine and Transplant Center, Cleveland Clinic Foundation, Cleveland, OH USA; Medtronic, Mansfield, MA USA; Interventional Pulmonology Fellowship Program, The University of Chicago Medicine, Chicago, IL USA; Department of Thoracic Oncology, Moffitt Cancer Center, Tampa, FL USA; Department of Medicine, Duke University School of Medicine, Durham, NC USA; Department of Radiation Oncology, The Ohio State University Wexner Medical Center, Columbus, OH USA; CVTSA/Inova Health System, Fairfax Hospital, Falls Church, VA USA; Division of Thoracic Surgery and Interventional Pulmonology, Beth Israel Deaconess Medical Center, Harvard Medical School, 185 Pilgrim Road, Deaconess Building, Suite 201, Boston, MA 02215 USA

**Keywords:** Bronchoscopy, Electromagnetic navigation, Image-guided biopsy, Lung cancer, Lung neoplasms, Neoplasm staging, Solitary pulmonary nodule, superDimension

## Abstract

**Background:**

Electromagnetic navigation bronchoscopy (ENB) procedures allow physicians to access peripheral lung lesions beyond the reach of conventional bronchoscopy. However, published research is primarily limited to small, single-center studies using previous-generation ENB software. The impact of user experience, patient factors, and lesion/procedural characteristics remains largely unexplored in a large, multicenter study.

**Methods/Design:**

NAVIGATE (Cli***n***ic***a***l E***v***aluation of superD***i***mension™ Navi***ga***tion System for Elec***t***romagn***e***tic Navigation Bronchoscopy) is a prospective, multicenter, global, cohort study. The study aims to enroll up to 2,500 consecutive subjects presenting for evaluation of lung lesions utilizing the ENB procedure at up to 75 clinical sites in the United States, Europe, and Asia. Subjects will be assessed at baseline, at the time of procedure, and at 1, 12, and 24 months post-procedure. The pre-test probability of malignancy will be determined for peripheral lung nodules. Endpoints include procedure-related adverse events, including pneumothorax, bronchopulmonary hemorrhage, and respiratory failure, as well as quality of life, and subject satisfaction. Diagnostic yield and accuracy, repeat biopsy rate, tissue adequacy for genetic testing, and stage at diagnosis will be reported for biopsy procedures. Complementary technologies, such as fluoroscopy and endobronchial ultrasound, will be explored. Success rates of fiducial marker placement, dye marking, and lymph node biopsies will be captured when applicable. Subgroup analyses based on geography, demographics, investigator experience, and lesion and procedure characteristics are planned.

**Discussion:**

Study enrollment began in April 2015. As of February 19, 2016, 500 subjects had been enrolled at 23 clinical sites with enrollment ongoing. NAVIGATE will be the largest prospective, multicenter clinical study on ENB procedures to date and will provide real-world experience data on the utility of the ENB procedure in a broad range of clinical scenarios.

**Trial registration:**

ClinicalTrials.gov NCT02410837. Registered 31 March 2015.

**Electronic supplementary material:**

The online version of this article (doi:10.1186/s12890-016-0228-y) contains supplementary material, which is available to authorized users.

## Background

Lung cancer is the leading cause of cancer death among both men and women in developed countries globally. An estimated 1.8 million new cases of lung and airway cancers occurred worldwide in 2012 [1].

Rapid and precise diagnosis of suspicious lesions is crucial to determine the optimal treatment for abnormalities in the lung found on chest computed tomography (CT) imaging. According to the American Cancer Society’s 2015 estimates, the 5-year survival rate for distant (metastasized) lung cancer is 4 %, compared to 27 % for regional cancer and 54 % for localized cancer. However, few lung cancers are diagnosed at an early stage when more patients are amenable to curative treatment options [2].

The National Lung Screening Trial [3] clearly demonstrated the efficacy of screening in a defined high-risk subset of the population. While the results and their generalizability can be questioned, the trial established the utility of low-dose CT scanning over conventional chest radiography. In addition, declining costs of technology, greater availability of CT imaging, lower radiation dosing, and increased awareness all have led to more CT scans of the chest. These scans will inevitably identify abnormalities for which intervention may be necessary. Current diagnostic options include image-guided needle biopsy, surgery, and more recently, electromagnetic navigation bronchoscopy.

ENB is an image-guided approach that uses 3D-reconstructed CT-scan and sensor location technology to guide a steerable endoscopic probe to peripheral lung lesions that may be beyond the reach of conventional bronchoscopes [4, 5], potentially aiding in diagnosis of lung cancer at an early stage of disease. This technology will also allow diagnosis of pulmonary metastases or benign diseases, such as infectious lung lesions or granulomatous disease, without surgical incisions.

Table 1 summarizes 24 original research articles published to date on the use of ENB procedures to aid in the diagnosis of peripheral lung lesions [6–29], most of which have been summarized in several meta-analyses [30–32]. In the most recent meta-analysis, the pooled sensitivity, specificity, positive likelihood ratio, negative likelihood ratio, and diagnostic odds ratio were 82 %, 100 %, 19.36, 0.23, and 97.62, respectively [32]. The pooled pneumothorax rate was approximately 3 %, of which approximately 1.6 % required chest tube insertion [30].

**Table 1:** Prior clinical studies on ENB to aid in the diagnosis of peripheral lung lesions

First author and year	Study design	Number of centers	Number of subjects	Length of follow-up	Diagnostic yield aided by ENB procedures (%)	Pneumothorax (%)
Becker 2005 [6]	Prospective, single-arm	Single-center	29	N.R.	69.0	3.4
Gildea 2006 [7]	Prospective, single-arm	Single-center	58	Mean 10.5 months	74.1	3.5
Schwarz 2006 [8]	Prospective, single-arm	Single-center	13	N.R.	69.2	0.0
Eberhardt 2007a [9]	Prospective, RCT (EBUS only, ENB only, or combined)	Multi-center (2)	39 (ENB alone) 40 (ENB + EBUS)	N.R.	59.0 (ENB alone) 87.5 (ENB + EBUS)	5.0
Eberhardt 2007b [10]	Prospective, single-arm	Multi-center (2)	89	N.R.	67.4	2.2
Makris 2007 [11]	Prospective, single-arm	Single-center	40	N.R.	62.5	7.5
Wilson 2007 [12]	Retrospective, single-arm	Single-center	222	6 – 18 months	59.9	1.2
Bertoletti 2009 [13]	Prospective, single-arm	Single-center	53	>18 months	77.4	4.0
Lamprecht 2009 [14]	Retrospective, single-arm	Single-center	13	N.R.	76.9	0.0
Eberhardt 2010 [15]	Prospective, single-arm	Single-center	53	N.R.	75.5	1.9
Seijo 2010 [16]	Prospective, single-arm	Single-center	51	N.R.	66.7	0.0
Mahajan 2011 [17]	Retrospective, single-arm	Single-center	48	N.R.	77.0	10.2
Brownback 2012 [18]	Retrospective, single-arm	Single-center	55	N.R.	74.5	0.0
Jensen 2012 [19]	Retrospective, single-arm	Multi-center (5)	92	≥6 months	65.2	3.3
Lamprecht 2012 [20]	Prospective, single-arm	Single-center	112	N.R.	83.9	1.8
Pearlstein 2012 [21]	Retrospective, single-arm	Single-center	101	2 years	85.1	5.8
Balbo 2013 [22]	Retrospective, single-arm	Single-center	40	N.R.	70.7	0.0
Karnak 2013 [23]	Prospective, single-arm	Single-center	35	≥2 years	91.4	3.9
Khan 2013 [24]	Prospective, single-arm	Single-center	24	N.R.	75.0	0.0
Mohanasundaram 2013 [25]	Retrospective, single-arm	Single-center	41	≤2 years	89.4	13.0
Loo 2014 [26]	Retrospective, single-arm	Single-center	40	N.R.	94.0	0.0
Odronic 2014 [27]	Retrospective, single-arm	Single-center	91	1 year	85.7	5.3
Bowling 2015 [28]	Retrospective, single-arm	Single-center	107	≥18 months	73.6	2.5
Ost 2015 [29]	Retrospective	Multi-center	39 (ENB ^a^ alone) 227 (ENB ^a^ + EBUS)	1 year	38.5 (ENB alone) 47.1 (ENB + EBUS)	1.7 %

Acronyms: *EBUS* endobronchial ultrasound, *ENB* electromagnetic navigation bronchoscopy, *RCT* randomized controlled trial

^a^Includes 252 cases using the superDimension™ navigation system, 8 cases using other electromagnetic navigation methods, and 14 cases using CT fluoroscopy. More than one method was used in some patients. In the publication by Ost et al., [29], diagnostic yield included only those cases with a specific malignant or benign diagnosis. If only inflammatory tissue or lymphocytes was obtained, the procedure was considered nondiagnostic

ENB procedures can also be used to aid in the diagnosis of mediastinal lymphadenopathy [33], as a localization tool for dye marking prior to video- or robotic-assisted thoracoscopic surgery [34], or to place fiducial markers prior to stereotactic body radiation therapy [35], all in a single procedure.

Despite these promising results, outcomes following ENB procedures are widely varied and most evidence to date is based on single-center analyses. Of the 24 published original studies on the use of ENB procedures to aid in the diagnosis of peripheral lung lesions, only 4 studies were multicenter [9, 10, 19, 29] and only 5 enrolled greater than 100 subjects [12, 20, 21, 28, 29] (see Table 1). The impact of user experience, patient risk factors, and lesion/procedural characteristics remains largely unexplored in a large multi-center study. The lack of clearly defined performance outcomes, varied definitions of diagnostic yield and accuracy, inconsistent use of complementary techniques, such as radial endobronchial ultrasound (EBUS), use of older ENB software versions, and variability in lesions targeted and biopsy methods also challenge the interpretation of results across studies. In addition, the full profile of how ENB procedures are used in daily practice (e.g., peripheral versus central lesions, lymph node sampling, dye marking, and fiducial marker placement) is not well understood. As ENB usage increases and is adopted by community centers with a wide range of user experience levels, the full safety profile across diverse clinical scenarios must be well understood, particularly with respect to the patient, lesion, and procedural factors associated with performance outcomes.

NAVIGATE (Cli***n***ic***a***l E***v***aluation of superD***i***mension™ Navi***ga***tion System for Elec***t***romagn***e***tic Navigation Bronchoscopy) aims to enroll up to 2,500 consecutive subjects at up to 75 sites worldwide. When complete, NAVIGATE will be the largest prospective, multicenter clinical study of ENB procedures to date. The objective of this observational study is to evaluate the safety profile and outcomes following ENB procedures conducted for suspicion of cancer or other diseases in lung lesions, for lymph node biopsy, or for dye marking or fiducial marker placement. The study is designed to capture the real-world experience of ENB use across heterogeneous sites and geographies, without restriction on ancillary tools or procedures. The 24-month follow-up will also allow an assessment of long-term diagnostic accuracy and the correlation between stage of diagnosis and short-term survival rates.

## Methods/Design

### Ethical compliance

This study is being conducted in accordance with the Declaration of Helsinki and all local regulatory requirements. As of February 19, 2016, 23 clinical sites in the United States and Europe had been activated into study (see Additional file 1 for the list of activated sites to date). The protocol was approved by the institutional review board of each participating site. Additional site selection is ongoing. Institutional review board approval will be obtained for each newly activated site prior to subject enrollment. Inclusion of up to 75 clinical sites is prespecified per protocol. The study was registered under ClinicalTrials.gov identifier NCT02410837 on March 31, 2015, which will be updated as new sites are activated. All subjects have provided or will provide written informed consent prior to enrollment.

### Trial design and subject eligibility

NAVIGATE is a prospective, single-arm, multicenter, post-market, observational study of ENB procedure use. The study aims to enroll up to 2,500 subjects at up to 75 clinical sites worldwide, including the United States, Europe, China, Korea, and Japan. To ensure heterogeneity of the data, a maximum enrollment per site will be specified for each region, approximately 75 subjects per site in the US, with other regions to be determined.

All consecutive patients over the age of 18, who are candidates for an elective ENB procedure and who present for evaluation of lung lesions, are eligible for enrollment. Exclusion criteria are: (1) the patient is unable or unwilling to provide informed consent or to comply with study follow-up schedule; (2) the patient has participated in an investigational drug or device research study within 30 days of enrollment that would interfere with this study; and (3) the patient is pregnant or nursing.

### Test device

The superDimension™ navigation system, software version 6.0 or higher (Medtronic, Minneapolis, MN), will be used to conduct all ENB procedures [4–6, 8].

### Pre-procedure assessments

A full list of subject demographics, lesion characteristics, and pre-procedure data to be collected is included in Table 2.

**Table 2:** Study assessments

Subject demographics
• Age
• Sex
• Ethnicity
• Race
Subject medical history and baseline status
• Prior invasive lung procedures and surgeries
• Lung function and diffusing capacity
• Antithrombotic medication current and prior status, including duration of any discontinuation
• Subject risk factors
• Pre-procedure probability of malignancy
• Quality of life (EQ-5D)
Lesion characteristics
• Size
• Location
• Presence of bronchus sign on computerized tomography (CT)
• Lung zone (peripheral, middle, and proximal thirds)
• Visibility on fluoroscopy (if applicable)
• Positron emission tomography (PET)-positive (yes/no)
• Associated lymphadenopathy
• Distance to closest fissure
• Distance from lesion to pleura
• Preprocedure probability of malignancy (investigator assessment)
Procedural assessments
• Indication for procedure
• Anesthesia type
• Catheter type
• Procedure duration
• Imaging used (fluoroscopy, PET, radial endobronchial ultrasound [EBUS])
• Ability to successfully navigate to lesion
• Use of associated tools and type (e.g., access tools, biopsy forceps, cytology brush, aspiration needle)
• Number of lesions biopsied
• Number of lymph nodes biopsied (if applicable)
• Placement of fiducial markers (if applicable), type used, indication, and status at follow-up imaging
• Surgical resection, including use of dye marker, type used, and adequacy for surgical resection
• Diagnosis by both cytologic rapid on-site evaluation (ROSE) and pathology
• Cancer type (primary or metastatic), if applicable
• Cancer stage, if applicable
• Adequacy of sample for molecular testing and mutation type (if applicable)
• Lymph node number, station, size, and success in obtaining sample (if applicable)
• Number and type of repeat electromagnetic navigational bronchoscopy (ENB) procedures or other biopsies
• Other health services (e.g., imaging, transfusion, surgery, emergency room admission, prescriptions) received during admission for index procedure
• Hospital admission duration
• Adverse events, action taken, relationship to device, and outcome
Follow-up assessments
• Subject satisfaction (at 1-month follow-up only)
• Subject quality of life (EQ-5D) at all follow-up visits
• ENB Productivity and Activity Questionnaire (ENB-PAQ) at 1 month visit
• All health services (e.g., imaging, transfusion, surgery, emergency room admission, prescriptions) received since last visit
• All healthcare services related to lung health since index procedure (e.g., primary care and specialist visits, hospital, emergency room, oncology, radiology, pain management).
• All therapeutic and diagnostic procedures and diagnoses related to lung health since last visit
• Adverse events, action taken, relationship to device, and outcome

In addition, to assess the value of the ENB procedure as a decision-making tool, the pre-procedure probability of malignancy will be assessed for each subject being evaluated for diagnosis of a pulmonary nodule [36, 37]. Probability will be captured both by the investigator’s subjective estimation of pretest probability of malignancy and a mathematical model, to allow comparison between the two methods. The probability model employs a multiple regression equation including the following factors: (1) subject age; (2) smoking history; (3) history of extrathoracic malignancy; (4) nodule size; (5) nodule border characteristics; and (6) nodule location (see Additional file 2 for the full equation). See Additional file 3 for the definition of peripheral lung lesions. 

### Procedures

The ENB procedure will be performed per product instructions and the institution’s standard practice. All complementary devices and procedures (e.g., catheter type, biopsy tools, access tools, fiducial placement or dye marking, and concomitant imaging, such as radial EBUS) are at the discretion of the investigator and are not mandated per the study protocol. All complementary procedures and devices will be captured (Table 2).

### Primary and secondary endpoints

This post-market study is intended to capture clinical outcomes related to the real-world use of ENB procedures, including but not limited to peripheral lesion biopsies, lymph node biopsies, fiducial marker placement for radiation therapy, and tumor marking for diagnostic and therapeutic surgery.

The primary endpoint is the incidence of pneumothorax related to the ENB index procedure rated as Grade 2 or higher according to the Common Terminology Criteria for Adverse Events (CTCAE) scale (Table 3) [38]. The primary endpoint was chosen as a comprehensive endpoint that would be applicable to all ENB procedures. The secondary endpoints will be assessed as applicable to the ENB procedure being conducted.

**Table 3:** Study definitions

Bronchopulmonary hemorrhage
A disorder characterized by bleeding from the bronchial wall and/or lung parenchyma. Degree of severity will be classified according to Common Terminology Criteria for Adverse Events (CTCAE) grade [38].The incidence of bronchopulmonary hemorrhage related to ENB index procedure rated as Grade 2 or higher according to CTCAE scale will be captured and reported:
• Grade 2: Moderate symptoms; medical intervention indicated
• Grade 3: Transfusion, radiologic, endoscopic, or operative intervention indicated (e.g., hemostasis of bleeding site)
• Grade 4: Life-threatening respiratory or hemodynamic compromise; intubation or urgent intervention indicated
• Grade 5: Death
Bronchopulmonary hemorrhage will also be reported based on a newly validated airway bleeding scale (E. Folch and S. Khandhar, unpublished personal communication; publication in progress).
Diagnostic yield
Diagnostic yield of the ENB procedure will be calculated on a per-subject basis out of all subjects in whom a diagnostic biopsy is attempted and is defined as the proportion of subjects in whom the ENB procedure yielded a definitive diagnosis. Accuracy of all diagnoses aided by the ENB procedure, as well as nondiagnostic cases, will evaluated based on 2-year clinical follow-up (see below).
Diagnostic accuracy
Sensitivity, specificity, positive predictive value (PPV), and negative predictive value (NPV) will be calculated as the accuracy of the ENB-aided diagnosis (based on final pathology results) compared to the final 2-year diagnosis based on all available procedures and follow-up.
Where a = true positive, b = false positive, c = false negative, and d = true negative:
• Sensitivity: Probability that an ENB-guided biopsy will be positive when malignancy is present (true positive rate): = a/(a + c)
• Specificity: Probability that an ENB-guided biopsy will be negative when malignancy is not present (true negative rate): = d/(b + d)
• PPV: Probability that malignancy is present when an ENB-guided biopsy is positive: = a/(a + b)
• NPV: Probability that malignancy is not present when an ENB-guided biopsy is negative: = d/(c + d)
Lesion size
Defined as greatest diameter of the target lesion.
Navigation Accuracy
Distance between the tip of the locatable guide and the targeted lung lesion (not done for lymph nodes).
Navigation success
The proportion of cases in which the operator is able to successfully navigate to the lung target with ENB guidance, based on investigator self-assessment.
Navigation time
Total time that the locatable guide is used in the subject during the ENB procedure.
Peripheral lung lesion
A lesion that is located in the outer third of the lung and difficult to reach by traditional bronchoscopy (see Additional file 3, Peripheral Lung Lesion Definition).
Pneumothorax
A disorder characterized by abnormal presence of air in the pleural cavity resulting in the collapse of the lung. The primary endpoint is the incidence of pneumothorax related to the ENB index procedure rated as Grade 2 or higher. All grades will be captured as a secondary endpoint. Degree of severity will be classified according to CTCAE grade as follows:
• Grade 1: Asymptomatic; clinical or diagnostic observations only; intervention not indicated.
• Grade 2: Symptomatic; intervention indicated (e.g., tube placement without sclerosis).
• Grade 3: Sclerosis and/or operative intervention indicated; hospitalization indicated.
• Grade 4: Life-threatening consequences; urgent intervention indicated.
• Grade 5: Death.
Pneumothorax severity will also be reported based on a newly validated scale that takes into account variance in international standard of care for observation and intervention following asymptomatic pneumothorax and compared to the CTCAE criteria.
Procedure time
Time between initial introduction of the locatable guide into the body and final removal of the locatable guide.
Repeat biopsy
Additional biopsy or biopsies conducted after the ENB-guided index procedure, required due to insufficient tissue collection for diagnosis at the index procedure.
Respiratory failure
A disorder characterized by impaired gas exchange by the respiratory system resulting in hypoxemia and a decrease in oxygenation of the tissues that may be associated with an increase in arterial levels of carbon dioxide. Degree of severity will be classified according to CTCAE grade as follows:
• Grade 4: Life-threatening consequences; urgent intervention, intubation, or ventilatory support indicated.
• Grade 5: Death.
Stage
Lung cancer staging will be defined according to the Revised International System for Staging Lung Cancer (TNM) Classification System.
Tissue adequacy
Tissue adequacy is defined as the proportion of cases in which tissue obtained during the ENB index procedure is adequate for subtyping of lung cancer and molecular testing when appropriate

The following secondary endpoints will evaluated for all ENB index procedures:

1. Incidence of all pneumothoraces related to ENB index procedure
2. Incidence of bronchopulmonary hemorrhage related to ENB index procedure rated as Grade 2 or higher according to the CTCAE scale
3. Incidence of respiratory failure related to ENB index procedure rated as Grade 4 or higher according to the CTCAE scale
4. Subject health status and quality of life evaluated by EQ-5D-3 L questionnaire (Version 5.1, April 2015) [39, 40]
5. Subject self-reported satisfaction at 1-month post-procedure, including referral source, pre-procedure expectations, pain, willingness to undergo another ENB procedure, worker productivity, and willingness to recommend the ENB procedure to family and friends.
6. ENB procedure effect on subject productivity and activity using the ENB Productivity and Activity Questionnaire (ENB-PAQ) at 1-month visit.

The following secondary endpoints will be evaluated for all ENB index procedures performed for suspicion of cancer or other diseases in a lung lesion. See Table 3 for definitions.

7. Diagnostic yield
8. Sensitivity
9. Specificity
10. Positive predictive value
11. Negative predictive value
12. Repeat biopsy rate due to lack of diagnosis during ENB index procedure
13. Adequacy of sample for molecular testing and mutation type (if applicable)
14. Diagnosis
15. Stage at diagnosis (if applicable)

In addition, the following secondary endpoints will be evaluated for applicable procedures:

7. Success rate of accurate placement of fiducial markers demonstrated through follow-up imaging
8. Success rate of dye marking demonstrated by successful surgical resection
9. Success rate of obtaining lymph node biopsy to provide stage of diagnosis

In addition to the primary and secondary endpoints listed above, all adverse events related to the ENB procedure or associated tools will be captured and reported, including all incidences of death, pneumothorax, bronchopulmonary hemorrhage, and respiratory failure regardless of CTCAE grade. New scales have also been developed by the study investigators and validated by expert consensus (Delphi technique [41]) to provide more specific criteria for defining the incidence and severity of bronchopulmonary hemorrhage and pneumothorax in subjects undergoing transbronchial lung biopsies under flexible bronchoscopy. These scales will be evaluated for the first time in the NAVIGATE study and compared to the existing CTCAE scale (E. Folch and S. Khandhar, unpublished personal communication; publication in progress). Additional procedural and follow-up assessments are listed in Table 2.

### Follow-up

Subjects will be evaluated at baseline (within 30 days of the procedure), on the procedure day, and at 1 month, 12 months, and 24 months post-procedure. Follow-up will capture any repeat diagnostic procedures or new diagnoses on any lung nodule evaluated during the initial ENB index procedure, as well as healthcare utilization since the ENB index procedure (including healthcare visits, repeat ENB procedures, transthoracic biopsy, bronchoscopy, chemotherapy, brachytherapy, radiation therapy, surgical resection, and lymph node dissection). All lung nodules evaluated during the initial ENB index procedure will be followed for confirmation of ENB-aided diagnoses, including confirmation of initially negative or inconclusive results.

### Investigator training

All investigators must have completed the superDimension™ navigation system training course prior to enrolling subjects into the study. In order to capture the full breadth of ENB procedure usage at both high-volume and low-volume centers, investigators without extensive experience conducting ENB procedures will be allowed to perform a maximum of 5 “roll-in cases”.

Roll-in subjects will be considered study participants and will complete all protocol-required procedures and follow-up. However, data for the roll-in group will be analyzed separately from the remaining study population in an effort to detect any differences in outcomes related to the level of investigator experience.

### Statistics

No sample size calculations were conducted for this single-arm, observational study. All statistical analyses will be performed using Statistical Analysis System (SAS) for Windows (Version 9.2 or higher, SAS Institute Inc. Cary, NC) or other widely accepted statistical or graphical software.

Descriptive statistics will be used to present the data and to summarize the results. Discrete variables will be presented using frequency distributions and cross tabulations. Continuous variables will be summarized by presenting the number of observations (n), mean, standard deviation, median, minimum, and maximum values. For the primary endpoint, a 2-sided 95 % exact binomial confidence interval will also be provided.

In general, data analysis will be conducted on all subjects who are enrolled in the trial and undergo an ENB procedure. Analyses will be conducted on multiple subsets of study subjects based upon variables such as geography, demographics, investigator experience, lesion and procedure characteristics, and other variables as deemed appropriate. Multifactorial and subgroup analyses evaluating the impact of patient (e.g., medical and surgical history, risk profile), lesion (e.g., size, location), and procedural characteristics (e.g., rapid on-site evaluation use, tissue adequacy, anesthesia method, use of complementary technology) are planned.

Interim analyses are prespecified per protocol when the first 500, 1000, and 1500 subjects enrolled reach the 1-month and 1-year follow-up timepoints.

### Study organization

The sponsor will utilize a medical monitor to provide a medical review and adjudication of pre-specified adverse events in support of the protocol-defined endpoint data. The medical monitor is a qualified, board-certified physician who is not affiliated with an investigative center or the study sponsor.

In addition, a global Investigator Steering Committee will provide oversight of study conduct, data analysis, subject safety, and the publication of results in alignment with recommendations of the International Committee of Medical Journal Editors.

### Enrollment status

Enrollment began in April 2015 and is currently in progress. As of February 19, 2016, 500 subjects had been enrolled at 23 clinical sites in the United States and Europe. An interim analysis of the 1-month follow-up of this first cohort is planned for the fall of 2016, with a focus on patient and lesion characteristics and usage profile. Study enrollment is progressing on schedule to reach the planned number of approximately 2,500 subjects; however, final enrollment numbers are not guaranteed.

## Discussion

Current guidelines for the evaluation of patients suspected of having lung cancer recommend the least invasive method appropriate to the patient’s medical presentation, with flexible bronchoscopy as the first line approach for central lesions [42]. For peripheral lesions difficult to reach with conventional bronchoscopy, options include percutaneous transthoracic CT- or fluoroscopy-guided techniques or guided bronchoscopy. While transthoracic needle aspiration (TTNA) is preferred due to its high diagnostic yield, in a meta-analysis of 20 studies the mean pneumothorax rate was 25 % (range 4.2 %–60 %) [43].

Guided bronchoscopic methods include ENB, radial EBUS, and virtual bronchoscopy. A meta-analysis of guided bronchoscopic methods found that the weighted diagnostic yield of these techniques was higher than that of traditional bronchoscopy but lower than the typical yield of TTNA, with a wide variation among studies [31]. Radial EBUS has a low pneumothorax rate (approximately 1 %), similar to that of ENB. Like ENB, studies of radial EBUS-guided localization have reported varied outcomes dependent on user experience, with sensitivity for detection of malignancy ranging from 49 % to 88 % and a pooled sensitivity of 73 % [44]. Radial EBUS can be used in conjunction with ENB to confirm tumor location after initial navigation with ENB, with higher diagnostic yield than either procedure alone [9]. However, radial EBUS has been shown to have a lower diagnostic yield in lesions ≤20 mm in size [44]. Virtual bronchoscopy using an ultrathin bronchoscope, may also improve diagnostic yield when combined with radial EBUS, but is not widely available and is limited by the lack of a working channel for biopsy [45].

ENB procedures provide a minimally invasive, alternative method to access peripheral lung lesions and offer the potential for biopsy, fiducial marker placement, and/or dye marking in a single procedure. Access to smaller, more peripheral lesions with a low risk of pneumothorax may also allow diagnosis of lung cancer at an earlier stage. However, current research is limited mostly to small, single-center and single-operator studies, with diagnostic yield estimates generally ranging between 59 and 94 %. In contrast, one recent registry reported a diagnostic yield rate of only 38.5 % in 39 cases using ENB guidance alone and 47.1 % in 266 cases using ENB combined with EBUS, with overall diagnostic yield ranging from 33 % to 73 % across the 10 included centers [29]. Thus, the full picture of how ENB procedures are used in clinical practice across diverse sites and geographies remains unclear. By providing a large, heterogeneous dataset, the NAVIGATE study will address several important knowledge gaps and provide data on the real-world use of ENB procedures.

### Long-term data

Of the 24 published original studies on the use of ENB procedures to aid in the diagnosis of peripheral lung lesions presented in Table 1, fewer than half reported on patient outcomes beyond 1 year. Long-term data from the NAVIGATE study will elucidate the accuracy of ENB-aided diagnoses over time and provide valuable information on how that diagnosis and the stage of diagnosis may influence the patient’s treatment path.

### New data

The 3 published meta-analyses currently available on ENB procedures all draw largely from the same dataset [30–32]. NAVIGATE will provide an entirely unique dataset derived from a diverse patient population. Furthermore, because product versions and ancillary tools, as well as the way operators use those tools, are constantly evolving, NAVIGATE will provide an updated source of information that the medical community can use to evaluate this procedure to the present day. As technology progresses and new products are introduced, this will also be captured in NAVIGATE with the potential to examine the impact of technology iterations on performance.

### Generalizable data

With an enrollment cap of 75 subjects per site, NAVIGATE will collect data from a diverse array of sites, user experience levels, and geographies. More importantly, the study allows complete independence regarding user preferences and diagnostic modalities. While all ancillary biopsy tools, complementary imaging methods, and follow-up procedures will be captured and reported, NAVIGATE does not mandate the use of any specific procedure. Rather, NAVIGATE is designed simply to evaluate the clinical use of the ENB procedure in the medical community. The eligibility criteria for enrollment into NAVIGATE are intentionally broad to allow an unrestricted assessment of how is ENB used in everyday clinical practice in accordance with current guidelines. In particular, while ENB is typically thought of as a tool for the evaluation of peripheral lesions, the profile of ENB use for peripheral versus central lesions, primary cancers versus metastatic disease, and operable versus inoperable patients is not well known. NAVIGATE is designed to capture that information and increase our understanding of the population of patients in whom ENB is used and what patient profile is most appropriate for the ENB procedure versus alternative methods. NAVIGATE will also evaluate the usage and utility of ENB-aided samples for genetic testing, according to local practice requirements. This will provide current information to determine the value of ENB in this constantly changing field of knowledge.

### Standardized definitions

In addition to variations among user experience and patient characteristics, an important reason for the range in ENB-aided diagnostic yield across studies is simply the definition of diagnostic yield itself. Many studies lack clearly defined performance outcomes or differ in how indeterminate results are handled. NAVIGATE will use well-defined and widely accepted definitions of diagnostic yield, sensitivity, specificity, and negative and positive predictive value and evaluate the validity of those definitions across a large dataset.

NAVIGATE will also evaluate the validity of common definitions for pneumothorax and bronchopulmonary hemorrhage. In particular, because there is no internationally standardized scale to measure airway bleeding, NAVIGATE will pilot the use of a new scale developed by investigators in this study and validated by expert consensus (E. Folch and S. Khandhar, unpublished personal communication; publication in progress). This scale will be evaluated side-by-side with the internationally standardized and recognized CTCAE grading system [38]. Pneumothorax severity will also be evaluated by a newly validated expert consensus scale that takes into account variance in international standard of care for observation and intervention following asymptomatic pneumothorax and compared to the CTCAE criteria (E. Folch and S. Khandhar, unpublished personal communication).

### Stage migration

Only 15 % of all lung cancers are diagnosed at a localized stage when the cancer is more amenable to surgical resection for curative intent, which is typically associated with the highest chance of long-term survival [2]. Diagnosis at an earlier stage also results in significant cost savings [46]. With the ability to reach smaller, more peripheral lesions, ENB procedures may theoretically enable diagnosis of lung cancer at an earlier stage. A retrospective review of 286 cases of non–small-cell lung cancer in a single center demonstrated that there was a greater proportion of early-stage diagnoses after the introduction of ENB compared to before the introduction of ENB [47]. NAVIGATE will prospectively assess stage at diagnosis and other associated procedures within the follow-up timeframe to confirm this result in a larger, multicenter dataset.

### Healthcare utilization and economic profile

One criticism of ENB is that it is more time-consuming and expensive and requires more resources than other techniques [30]. However, other studies have reported that, despite higher acquisition cost, ENB procedures are slightly less expensive than TTNA due to higher complication costs with TTNA [48]. NAVIGATE will assess ENB costs, as well as downstream payments for follow-up procedures, to determine how ENB – and potentially an earlier diagnosis and lower pneumothorax rate – impacts long-term cost-effectiveness. NAVIGATE will also evaluate all healthcare services and procedures related to lung cancer diagnosis and treatment following the initial ENB index procedure through 2-year follow-up, for the purpose of defining the treatment pathway after the initial ENB-aided diagnosis.

### Predicting the probability of malignancy

With recent discussions on the value of screening for lung cancer, the number of patients with positive CT findings is likely to increase, particularly with the recommendations for low-dose CT screening for high-risk individuals by joint societies (e.g., Centers for Medicare and Medicaid Services, Unites States Preventive Services Task Force, National Comprehensive Cancer Network, American College of Chest Physicians, Society of Thoracic Surgeons) [49]. Published studies have reported that approximately 25 % of patients who undergo low-dose CT screening will have a positive finding [3], with up to 50 % in smokers over the age of 50 [50]. The anxiety and stress that patients experience from a positive CT finding can be further compounded by the development of a complication during the diagnostic workup. In the NLST study, 8.2 %–11.2 % of patients diagnosed with lung cancer were associated with having a major complication after an invasive procedure [3]. Thus, there is an increasing need for appropriate tools and information to guide physicians on how to handle positive findings based on the clinical estimation of risk. The clinician must evaluate the overall probability of malignancy in order to determine which patients can be safely observed (<10 % probability of malignancy) and which require immediate excision (>90 % probability of malignancy). For moderately sized or indeterminate lesions, the appropriate course is often unclear. NAVIGATE will provide information on which approach (watchful waiting with serial CT scans, tissue biopsy, or direct excision) proves most effective over time based on pre-procedure probability of malignancy and ENB findings. NAVIGATE will also compare physician-estimated pre-procedure probability of malignancy to a mathematical model that calculates risk based on patient age, smoking history, history of extrathoracic malignancy, nodule size, nodule border characteristics, and nodule location [36, 37]. This calculation tool has been proven equivalent to other estimation tools, requires few data points, is easy to use, and is globally available. An ancillary objective of the NAVIGATE study is to validate this tool in a larger dataset and compare the long-term accuracy of the probability of malignancy based on this calculation compared to the clinician’s pre-procedure estimate.

### Quality of life

Lung cancer patients are increasingly concerned with quality of life and loss of lung function following surgical resection. Physician and patient comfort levels and comorbidities must also be considered in every case. When warranted, minimally invasive diagnostic options may be preferred for patients with suspicious nodules and low probability of malignancy.

NAVIGATE will assess patient health status and quality of life using the EQ-5D-3 L questionnaire [39, 40], a standardized measure of health status over a 24-month period. In addition, assessments of patient satisfaction, healthcare utilization, and worker productivity will provide information on the impact of ENB on the continuum of care. Potential subgroup analyses might also examine how quality of life, patient satisfaction, and healthcare utilization differ based on the purpose of the original ENB procedure (e.g., primary tumor versus metastases, screening versus diagnosis, staging, marker placement, etc.) and the diagnosis.

### Conclusions

NAVIGATE will have a substantial impact on both the pulmonology and thoracic surgery communities. With up to 2,500 subjects planned, NAVIGATE will be approximately 10 times larger than the largest ENB study to date. The intent of the study is to investigate real-world utilization patterns and outcomes in a broad range of both academic and community centers, including evaluations of diagnostic yield and accuracy and of success rates of fiducial marker placement, dye marking, and lymph node biopsies. Additionally, subgroup analyses are planned based on geography, patient demographics and medical history, investigator experience, and lesion and procedure characteristics. This study will answer questions that remain largely unexplored in a large multi-center trial and will provide a wealth of information to the medical community regarding the full profile of ENB usage. In addition, clinical utility and outcomes identified in NAVIGATE will be instructive for the design of future comparative studies.

## Additional files

Additional file 1: Activated Sites as of February 19, 2016. (PDF 42 kb) Additional file 2: Calculated Probability of Malignancy. (PDF 34 kb) Additional file 3: Peripheral Lung Lesion Definition. (PDF 91 kb)

## Abbreviations

CT: computed tomography
CTCAE: common terminology criteria for adverse events
EBUS: endobronchial ultrasound
ENB: electromagnetic navigation bronchoscopy
ENB-PAQ: ENB productivity and activity questionnaire
TTNA: transthoracic needle aspiration

## References

[1] Torre LA, Bray F, Siegel RL, Ferlay J, Lortet-Tieulent J, Jemal A (2015). Global cancer statistics, 2012. CA Cancer J Clin.

[2] Siegel RL, Miller KD, Jemal A (2015). Cancer statistics, 2015. CA Cancer J Clin.

[3] Team National Lung Screening Trial Research, Aberle DR, Adams AM, Berg CD, Black WC, Clapp JD (2011). Reduced lung-cancer mortality with low-dose computed tomographic screening. N Engl J Med..

[4] Weiser TS, Hyman K, Yun J, Litle V, Chin C, Swanson SJ (2008). Electromagnetic navigational bronchoscopy: a surgeon’s perspective. Ann Thorac Surg.

[5] Reynisson PJ, Leira HO, Hernes TN, Hofstad EF, Scali M, Sorger H (2014). Navigated bronchoscopy: a technical review. J Bronchol Interv Pulmonol.

[6] Becker HD, Herth F, Ernst A, Schwarz Y (2005). Bronchoscopic biopsy of peripheral lung lesions under electromagnetic guidance: a pilot study. J Bronchol Interv Pulmonol.

[7] Gildea TR, Mazzone PJ, Karnak D, Meziane M, Mehta AC (2006). Electromagnetic navigation diagnostic bronchoscopy: a prospective study. Am J Respir Crit Care Med.

[8] Schwarz Y, Greif J, Becker HD, Ernst A, Mehta A (2006). Real-time electromagnetic navigation bronchoscopy to peripheral lung lesions using overlaid CT images: the first human study. Chest.

[9] Eberhardt R, Anantham D, Ernst A, Feller-Kopman D, Herth F (2007). Multimodality bronchoscopic diagnosis of peripheral lung lesions: a randomized controlled trial. Am J Respir Crit Care Med.

[10] Eberhardt R, Anantham D, Herth F, Feller-Kopman D, Ernst A (2007). Electromagnetic navigation diagnostic bronchoscopy in peripheral lung lesions. Chest.

[11] Makris D, Scherpereel A, Leroy S, Bouchindhomme B, Faivre JB, Remy J (2007). Electromagnetic navigation diagnostic bronchoscopy for small peripheral lung lesions. Eur Respir J.

[12] Wilson DS, Bartlett RJ (2007). Improved diagnostic yield of bronchoscopy in a community practice: combination of electromagnetic navigation system and rapid on-site evaluation. J Bronchol Interv Pulmonol.

[13] Bertoletti L, Robert A, Cottier M, Chambonniere ML, Vergnon JM (2009). Accuracy and feasibility of electromagnetic navigated bronchoscopy under nitrous oxide sedation for pulmonary peripheral opacities: an outpatient study. Respiration.

[14] Lamprecht B, Porsch P, Pirich C, Studnicka M (2009). Electromagnetic navigation bronchoscopy in combination with PET-CT and rapid on-site cytopathologic examination for diagnosis of peripheral lung lesions. Lung.

[15] Eberhardt R, Morgan RK, Ernst A, Beyer T, Herth FJ (2010). Comparison of suction catheter versus forceps biopsy for sampling of solitary pulmonary nodules guided by electromagnetic navigational bronchoscopy. Respiration.

[16] Seijo LM, de Torres JP, Lozano MD, Bastarrika G, Alcaide AB, Lacunza MM (2010). Diagnostic yield of electromagnetic navigation bronchoscopy is highly dependent on the presence of a bronchus sign on CT imaging: results from a prospective study. Chest..

[17] Mahajan AK, Patel S, Hogarth DK, Wightman R (2011). Electromagnetic navigational bronchoscopy: an effective and safe approach to diagnose peripheral lung lesions unreachable by conventional bronchoscopy in high-risk patients. J Bronchol Interv Pulmonol.

[18] Brownback KR, Quijano F, Latham HE, Simpson SQ (2012). Electromagnetic navigational bronchoscopy in the diagnosis of lung lesions. J Bronchol Interv Pulmonol.

[19] Jensen KW, Hsia DW, Seijo LM, Feller-Kopman DJ, Lamb C, Berkowitz D (2012). Multicenter experience with electromagnetic navigation bronchoscopy for the diagnosis of pulmonary nodules. J Bronchol Interv Pulmonol.

[20] Lamprecht B, Porsch P, Wegleitner B, Strasser G, Kaiser B, Studnicka M (2012). Electromagnetic navigation bronchoscopy (ENB): Increasing diagnostic yield. Respir Med.

[21] Pearlstein DP, Quinn CC, Burtis CC, Ahn KW, Katch AJ (2012). Electromagnetic navigation bronchoscopy performed by thoracic surgeons: one center’s early success. Ann Thorac Surg.

[22] Balbo PE, Bodini BD, Patrucco F, Della Corte F, Zanaboni S, Bagnati P (2013). Electromagnetic navigation bronchoscopy and rapid on site evaluation added to fluoroscopy-guided assisted bronchoscopy and rapid on site evaluation: improved yield in pulmonary nodules. Minerva Chir.

[23] Karnak D, Ciledag A, Ceyhan K, Atasoy C, Akyar S, Kayacan O (2013). Rapid on-site evaluation and low registration error enhance the success of electromagnetic navigation bronchoscopy. Ann Thorac Med.

[24] Khan AY, Berkowitz D, Krimsky WS, Hogarth DK, Parks C, Bechara R (2013). Safety of pacemakers and defibrillators in electromagnetic navigation bronchoscopy. Chest.

[25] Mohanasundaram U, Ho LA, Kuschner WG, Chitkara RK, Canfield J, Canfield LM (2013). The diagnostic yield of navigational bronchoscopy performed with propofol deep sedation. ISRN Endoscopy.

[26] Loo FL, Halligan AM, Port JL, Hoda RS (2014). The emerging technique of electromagnetic navigation bronchoscopy-guided fine-needle aspiration of peripheral lung lesions: promising results in 50 lesions. Cancer Cytopathol.

[27] Odronic SI, Gildea TR, Chute DJ (2014). Electromagnetic navigation bronchoscopy-guided fine needle aspiration for the diagnosis of lung lesions. Diagn Cytopathol.

[28] Bowling MR, Kohan MW, Walker P, Efird J, Ben OS (2015). The effect of general anesthesia versus intravenous sedation on diagnostic yield and success in electromagnetic navigation bronchoscopy. J Bronchol Interv Pulmonol.

[29] Ost DE, Ernst A, Lei X, Kovitz KL, Benzaquen S, Diaz-Mendoza J (2016). Diagnostic yield and complications of bronchoscopy for peripheral lung lesions. Results of the AQuIRE registry. Am J Respir Crit Care Med.

[30] Gex G, Pralong JA, Combescure C, Seijo L, Rochat T, Soccal PM (2014). Diagnostic yield and safety of electromagnetic navigation bronchoscopy for lung nodules: a systematic review and meta-analysis. Respiration.

[31] Wang Memoli JS, Nietert PJ, Silvestri GA (2012). Meta-analysis of guided bronchoscopy for the evaluation of the pulmonary nodule. Chest.

[32] Zhang W, Chen S, Dong X, Lei P (2015). Meta-analysis of the diagnostic yield and safety of electromagnetic navigation bronchoscopy for lung nodules. J Thorac Dis.

[33] Diken ÖE, Karnak D, Çiledağ A, Ceyhan K, Atasoy Ç, Akyar S (2014). Electromagnetic navigation-guided TBNA vs conventional TBNA in the diagnosis of mediastinal lymphadenopathy. Clin Respir J.

[34] Bolton WD, Howe H, Stephenson JE (2014). The utility of electromagnetic navigational bronchoscopy as a localization tool for robotic resection of small pulmonary nodules. Ann Thorac Surg.

[35] Mungal V, Sarsour RM, Siddiqui AM, Awadallah S, Bowling MR (2015). The utility of bronchoscopy for the placement of fiducial markers for stereotactic body radiotherapy. Clin Pulm Med..

[36] Folch E, Mazzone P. Evaluation of Solitary Pulmonary Nodules. Epocrates, an athenahealth company, 2014. https://online.epocrates.com/u/2921547/Evaluation+of+solitary+pulmonary+nodule. Accessed 4 June 2015.

[37] Swensen SJ, Silverstein MD, Ilstrup DM, Schleck CD, Edell ES (1997). The probability of malignancy in solitary pulmonary nodules. Application to small radiologically indeterminate nodules. Arch Intern Med.

[38] National Cancer Institute [Internet]. Cancer Therapy Evaluation Program Common Terminology Criteria for Adverse Events (CTCAE), v4.0. 2009. http://ctep.cancer.gov/protocolDevelopment/electronic_applications/ctc.htm#ctc_40. Accessed 13 February 2015.

[39] EuroQol G (1990). EuroQol--a new facility for the measurement of health-related quality of life. Health Policy.

[40] EuroQol Group. EQ-5D Version 5.1 [Internet], April 2015. EuroQol Research Foundation, Rotterdam, The Netherlands. http://www.euroqol.org/about-eq-5d.html. Accessed 13 February 2015.

[41] Diamond IR, Grant RC, Feldman BM, Pencharz PB, Ling SC, Moore AM (2014). Defining consensus: a systematic review recommends methodologic criteria for reporting of Delphi studies. J Clin Epidemiol.

[42] Rivera MP, Mehta AC, Wahidi MM (2013). Establishing the diagnosis of lung cancer: Diagnosis and management of lung cancer, 3rd ed: American College of Chest Physicians evidence-based clinical practice guidelines. Chest.

[43] Wiener RS, Wiener DC, Gould MK (2013). Risks of transthoracic needle biopsy: how high?. Clin Pulm Med.

[44] Steinfort DP, Khor YH, Manser RL, Irving LB (2011). Radial probe endobronchial ultrasound for the diagnosis of peripheral lung cancer: systematic review and meta-analysis. Eur Respir J.

[45] Oki M, Saka H, Ando M, Asano F, Kurimoto N, Morita K (2015). Ultrathin bronchoscopy with multimodal devices for peripheral pulmonary lesions. A randomized trial. Am J Respir Crit Care Med.

[46] Pyenson B, Henschke C, Yip R, Dec E, Yankelevitz D (2014). Offering lung cancer screening to high-risk medicare beneficiaries saves lives and is cost-effective: an actuarial analysis. Am Health Drug Benefits.

[47] Brown C, Ben-Or S, Walker P, Bowling M (2016). The impact of electromagnetic navigational bronchoscopy on a multidisciplinary thoracic oncology program. J Natl Compr Canc Netw.

[48] Deppen SA, Davis WT, Green EA, Rickman O, Aldrich MC, Fletcher S (2014). Cost-effectiveness of initial diagnostic strategies for pulmonary nodules presenting to thoracic surgeons. Ann Thorac Surg.

[49] Centers for Disease Control and Prevention. Lung Cancer Screening Guidelines and Recommendations [Internet]. US Department of Health and Human Services, Atlanta. 2015. http://www.cdc.gov/cancer/lung/pdf/guidelines.pdf. Accessed 4 November 2015.

[50] MacMahon H, Austin JH, Gamsu G, Herold CJ, Jett JR, Naidich DP (2005). Guidelines for management of small pulmonary nodules detected on CT scans: a statement from the Fleischner Society. Radiology.

