# Supplementary material for: Design of a prospective, multicenter, global, cohort study of electromagnetic navigation bronchoscopy
Source: BMC Pulm Med. 2016 Apr 26;16:60. doi: 10.1186/s12890-016-0228-y (PMC4845335; doi:10.1186/s12890-016-0228-y)
Supplement: Additional file 1: — Activated Sites as of February 19, 2016. (PDF 42 kb) [file 12890_2016_228_MOESM1_ESM.pdf]

Additional File 1, Activated Sites as of February 19, 2016

As of February 19, 2016, 23 clinical sites in the United States and Europe had been activated into the NAVIGATE study. Additional site selection and activation is ongoing. The study protocol has prespecified up to 75 clinical sites in the United States, Europe, and Asia as needed to meet a final enrollment of up to 2,500 subjects. As of February 2016, study enrollment was progressing on schedule to reach the planned number of approximately 2,500 subjects; however, final enrollment and site numbers may change.

	**Site Name**	**City**	**State or Country**	**Region**	**Principal Investigator**
1	Pulmonary and Critical Care Associates of Baltimore	Baltimore	MD	USA	Krimsky, William
2	Pulmonary & Sleep of Tampa Bay	Brandon	FL	USA	Zanchi, Dragos
3	Virtua Medical Group, PA	Marlton	NJ	USA	Sztejman, Eric
4	Pinehurst Medical Center	Pinehurst	NC	USA	Pritchett, Michael
5	The University of Chicago	Chicago	IL	USA	Murgu, Septimiu
6	Providence Health Center	Waco	TX	USA	Murillo, Borris
7	University of Rochester	Rochester	NY	USA	Nead, Michael
8	Blount Memorial Hospital	Maryville	TN	USA	LeMense, Greg
9	Cancer Treatment Centers of America	Newnan	GA	USA	Bechara, Rabih
10	Penn Highlands Healthcare - DuBois	DuBois	PA	USA	Bansal, Sandeep
11	Duke University	Durham	NC	USA	Wahidi, Momen
12	Charleston Area Medical Center	Charleston	WV	USA	Takubo, Tom
13	East Texas Medical Center	Tyler	TX	USA	Gass, G. David
14	Carolinas Healthcare System	Charlotte	NC	USA	Singh, Jaspal
15	University of Cincinnati Physicians Company LLC	Cincinnati	OH	USA	Benzaquen, Sadia
16	Vanderbilt University Medical Center	Nashville	TN	USA	Rickman, Otis
17	University of Alabama at Birmingham	Birmingham	AL	USA	Minnich, Douglas
18	Pulmonary Associates of Mobile, PC	Mobile	AL	USA	Ellis, Blesilda
19	AKH Linz	Linz	Austria	Europe	Lamprecht, Bernd
20	Gunderson Lutheran Medical Foundation, Inc.	LaCrosse	WI	USA	Mattingley, Jennifer
21	University Hospitals of Case Medical Center	Cleveland	OH	USA	Linden, Philip
22	East Carolina University	Greenville	NC	USA	Bowling, Mark
23	University of Michigan Health Systems	Ann Arbor	MI	USA	Arenberg, Douglas
