# Supplementary material for: Design of a prospective, multicenter, global, cohort study of electromagnetic navigation bronchoscopy
Source: BMC Pulm Med. 2016 Apr 26;16:60. doi: 10.1186/s12890-016-0228-y (PMC4845335; doi:10.1186/s12890-016-0228-y)
Supplement: Additional file 2: — Calculated Probability of Malignancy. (PDF 34 kb) [file 12890_2016_228_MOESM2_ESM.pdf]

Additional File 2, Calculated Probability of Malignancy

The pre-procedure probability of malignancy will be calculated for each patient and compared to investigator-determined probability and long-term outcomes. The probability of malignancy is calculated based on the patient's clinical profile and radiographic findings. A multiple regression model has been developed that includes the following factors: (1) patient age; (2) smoking history; (3) history of extrathoracic malignancy; (4) nodule size; (5) nodule border characteristics; and (6) nodule location.

The equation is calculated as follows:

Probability of malignancy = e^x/(1 + e^x).

Where:

x = -6.8272 + (0.0391 * "age") + (0.7917 * "cigarettes") + (1.3388 * "cancer") + (0.1274 * "diameter") + (1.047 * "spiculation") + (0.7838 * "upper lobe")

e	the mathematical constant (Euler's number)
^	to the power of
"age"	patient's age in years
"cigarettes"	1 if current or former smoker, otherwise 0
"cancer"	1 if patient has history of extrathoracic cancer diagnosed >5 years before presentation, otherwise 0
"diameter"	diameter of the solitary pulmonary nodule in mm
"spiculation"	1 if spiculation is present, otherwise 0
"upper lobe"	1 if the nodule is located in the upper lobe, otherwise 0

References

1. Swensen SJ, Silverstein MD, Ilstrup DM, et al. The probability of malignancy in solitary pulmonary nodules. Application to small radiologically indeterminate nodules. Arch Intern Med 1997;157:849-855.

2. Folch E, Mazzone P. Evaluation of Solitary Pulmonary Nodules. [Epocrates, an athenahealth company Web Site] September 26, 2014. Available at https://online.epocrates.com/u/2921547/Evaluation+of+solitary+pulmonary+nodule. Accessed June 4, 2015.
