# Supplementary material for: Design of a prospective, multicenter, global, cohort study of electromagnetic navigation bronchoscopy
Source: BMC Pulm Med. 2016 Apr 26;16:60. doi: 10.1186/s12890-016-0228-y (PMC4845335; doi:10.1186/s12890-016-0228-y)

Additional File 3, Peripheral Lung Lesion Definition

For the purposes of NAVIGATE data collection, a peripheral lung lesion is defined as a lesion that is located in the outer third of the lung and difficult to reach by traditional bronchoscopy, in accordance with the following visual aid:

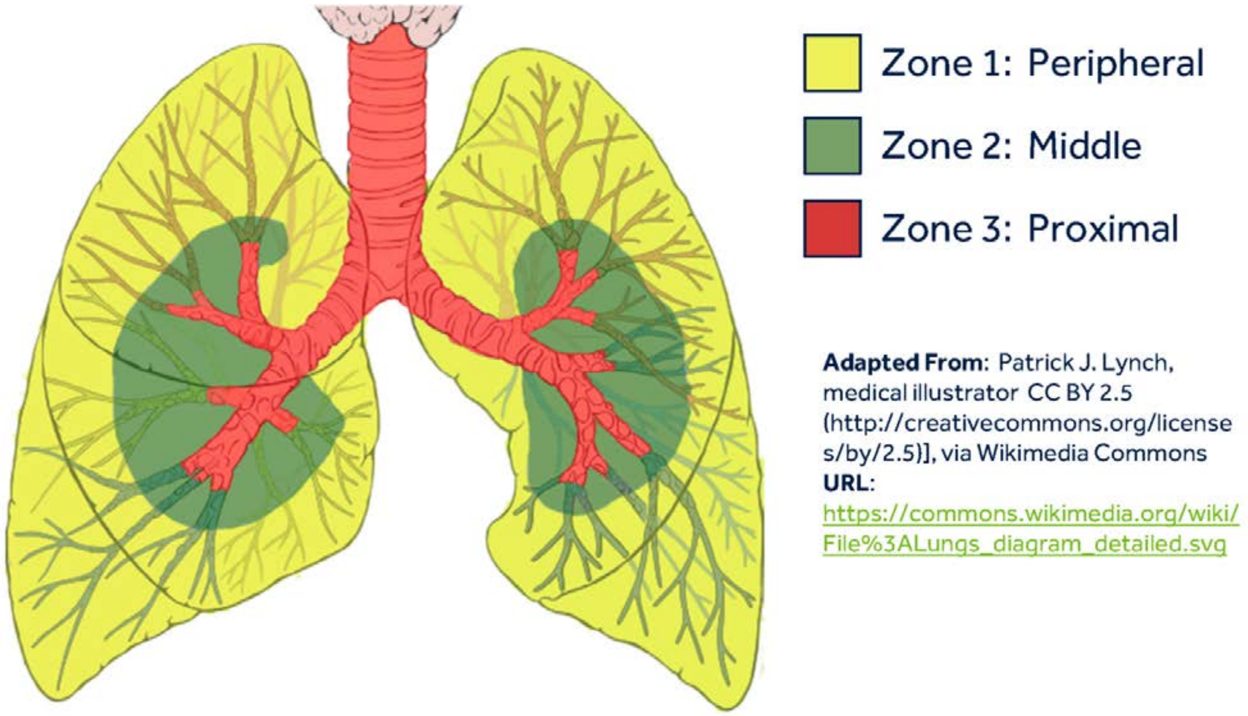

Supplement: Additional file 3: — Peripheral Lung Lesion Definition. (PDF 91 kb) [file 12890_2016_228_MOESM3_ESM.pdf]
